# Supplementary material for: Combined Transcriptomics and Metabolomics Uncover the Potential Mechanism of Plant Growth-Promoting Rhizobacteria on the Regrowth of Leymus chinensis After Mowing
Source: Int J Mol Sci. 2025 Jan 10;26(2):565. doi: 10.3390/ijms26020565 (PMC11766401; doi:10.3390/ijms26020565)
Supplement: Supplementary file 1 [file ijms-26-00565-s001.zip › ijms-3359946-supplementary.pdf]

Figures

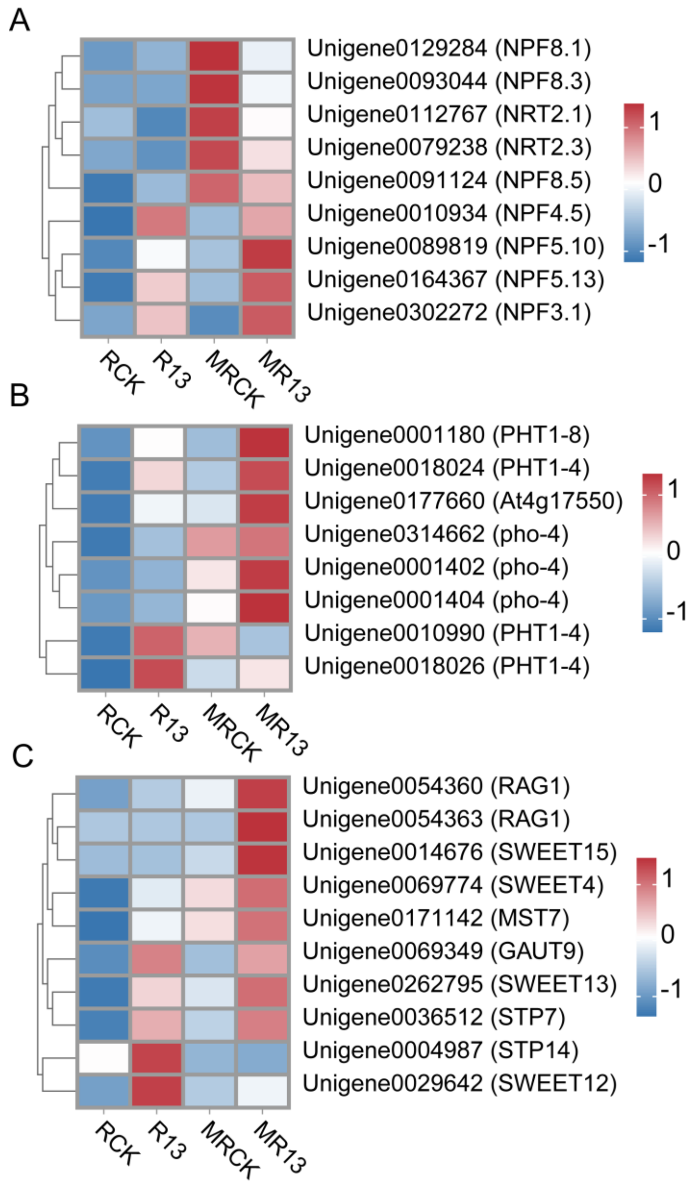

Figure S1 Expression of genes involved in nitrate transport (A), phosphate transport (B) and sugar transport (C).

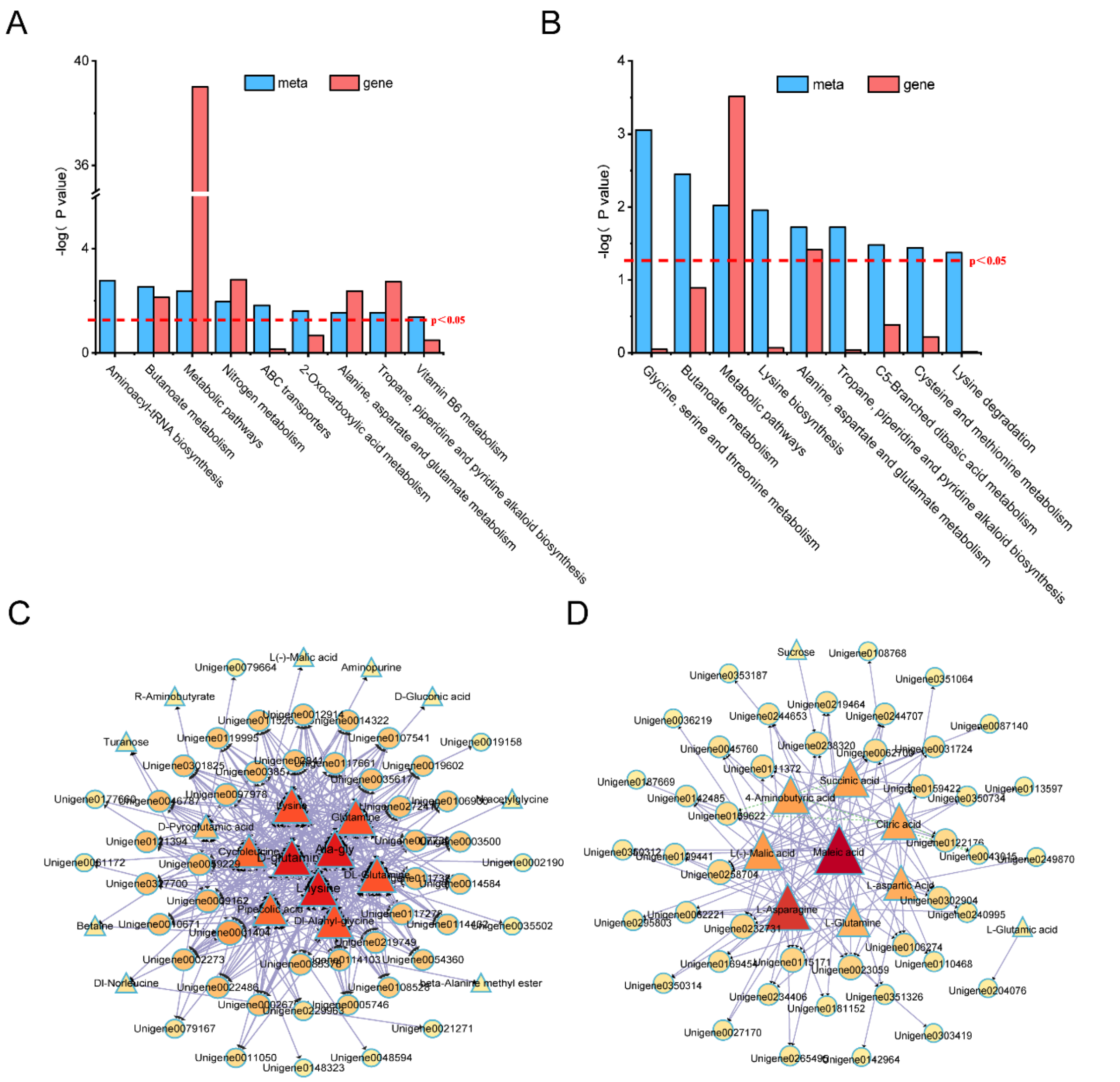

Figure S2 Integrated transcriptome and metabolome analysis. (A, B) KEGG pathway analysis of DEGs and DEMs in RCK vs. R13 and MRCK vs. MR13. (C) Co-expression network analysis of the 360 DEGs and 28 DEMs (correlation coefficient >0.95, $P<0.05$). (C) Co-expression network analysis of DEGs and DEMs in key metabolic pathways identified by transcriptome and metabolome association analyzed (Pearson correlation coefficient > 0.95 and p-value < 0.05). Network shows numerous connections between amino acid related DEMs and DEGs. Purple solid and green dotted lines are positive and negative interactions, respectively, between DEGs and DEMs. Triangular nodes indicate DEMs, and circular nodes indicate DEGs and. The color ranges from yellow to red, and the dots from small to large represent the degree of connectivity from low to high.

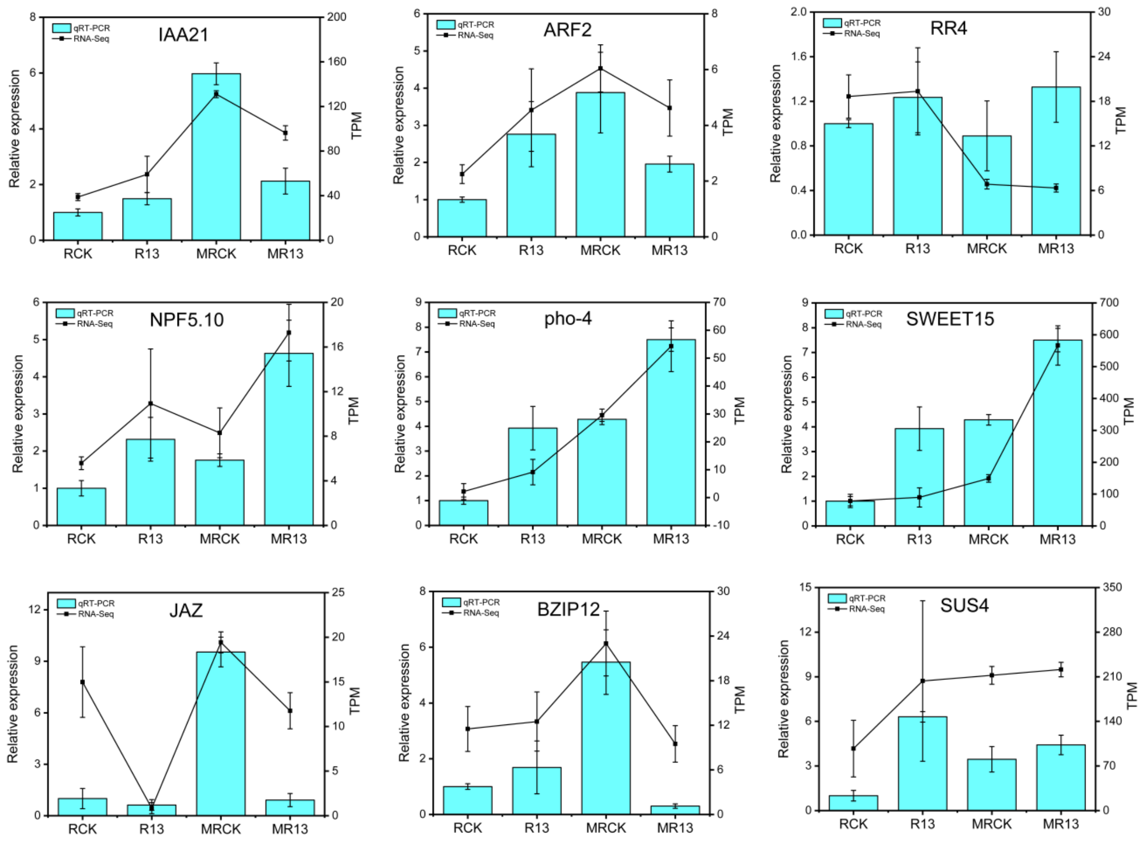

Figure S3 Transcriptional levels of genes related to nitrate transport, phosphate transport, sugar transport, starch and sucrose metabolism, alanine, aspartate and glutamate metabolism, plant hormone signal transduction were analyzed by qRT-PCR assay.

Table S1 Growth promoting characterization of *Pantoea eucalypti* B13

Growth-promoting characterization	Means±SE
Phosphate solubilization production (μg/mL)	107.38±8.80
IAA content (μg/mL)	29.41±6.21
Nitrogenase activity (IU/L)	186.38±6.36

Table S2 Quality statistics of RNA sequencing data.

Sample	Raw Reads	Clean Reads	Clean Data(bp)	Q20(%)	Q30(%)	GC Content(%)
RCK-1	37978752	37805664 (99.54%)	5661125053	5529010307 (97.67%)	5286614387 (93.38%)	3016745032 (53.29%)
RCK-2	37278080	37106470 (99.54%)	5556970968	5416899912 (97.48%)	5170006987 (93.04%)	2961841210 (53.30%)
RCK-3	40881188	40671116 (99.49%)	6091118075	5910373839 (97.03%)	5607881179 (92.07%)	3235145143 (53.11%)
R13-1	36705476	36546026 (99.57%)	5474357116	5330128895 (97.37%)	5078481200 (92.77%)	2914075656 (53.23%)
R13-2	44363612	44153374 (99.53%)	6612917358	6423511048 (97.14%)	6102555077 (92.28%)	3498751425 (52.91%)
R13-3	45833078	45648158 (99.60%)	6838727001	6671541066 (97.56%)	6372275063 (93.18%)	3580470922 (52.36%)
MRCK-1	36692636	36548632 (99.61%)	6838727001	6671541066 (97.56%)	6372275063 (93.18%)	3580470922 (52.36%)
MRCK-2	36424640	36310874 (99.69%)	5438093553	5301984653 (97.50%)	5062527731 (93.09%)	2897550335 (53.28%)
MRCK-3	42413742	42217028 (99.54%)	6322399047	6148927633 (97.26%)	5851729760 (92.56%)	3392112390 (53.65%)
MR13-1	4457933 (99.60%)	44400528 (99.60%)	6649486757	6475525085 (97.38%)	6170928730 (92.80%)	3587364283 (53.95%)
MR13-2	36325402	36178612 (99.60%)	5418419265	5283764349 (97.51%)	5046012193 (93.13%)	2910595668 (53.72%)
MR13-3	37667910	37507136 (99.57%)	5617737654	5456426937 (97.13%)	5190790305 (92.40%)	2998681930 (53.38%)

Table S3 Information on primers used in the qPCR assay.

Gene	Primer orientation	Primer sequence (5' > 3')
pho-4	forward	CAAGTGGTCGTGGCCTGTAT
pho-4	reverse	TCAAAAGACCCCATCGAGCC
SWEET15	forward	CTTCGTCTTCGGGGTGGC
SWEET15	reverse	GATCACGTCTTCGTCGGCT

NPF5.10	forward	GGAGGGCCAACGACTAGAG
NPF5.10	reverse	ACCACTCCGGGTGACGC
IAA21	forward	AGCAGGCAGCAGTTCCAG
IAA21	reverse	CCCAAGCAATAACAGAGCCC
ARF2	forward	CCACATGTTCTGCAAGACGC
ARF2	reverse	GTGGACAGCTGTACGTGGAT
RR4	forward	GTTCTTCCTGAAGCCCGTGA
RR4	reverse	CAGTCACTCGTGATTCCGGT
BZIP12	forward	ACGGCGACGCTCCCAC
BZIP12	reverse	CGGGGATCCTAACGGCG
JAZ	forward	CTCCGCCTTGTCTTCCATCC
JAZ	reverse	AGAGGCGCGGAGATCGATAA
SUS4	forward	CGATCGGTGAACTCGCCTTT
SUS4	reverse	GAGCGGTACAGAGCAGATTC
Actin	forward	ATTGTGCTCAGTGGTGGGTCA
Actin	reverse	CCAATCCAAACACTGTACTTCCTC
